# *Neuroradiology* and the COVID-19 pandemic

**DOI:** 10.1007/s00234-021-02868-8

**Published:** 2021-12-03

**Authors:** Rüdiger von Kummer

**Affiliations:** Institute and Policlinic of Diagnostic and Interventional Neuroradiology, Universitätsklinikum Dresden, Dresden, Germany

A Happy and Healthy New Year to you, well-respected readers, authors, reviewers, editors, and *Neuroradiology* Editorial Board members.

Though 2022 has begun, we have yet to overcome the coronavirus disease 2019 (COVID-19) pandemic, particularly in regions where vaccines are unavailable or where people refuse vaccination because they do not trust evidence-based science. The clear regional association between high COVID-19 incidence and low vaccination rates demonstrates the impact of illogical and unscientific thinking on the frequency of COVID-19 victims. As scientists, we are challenged to refute irrational arguments and guide global thinking on the pandemic.

*Neuroradiology’s* chief aim is to disseminate state-of-the-art scientific information in neuromedicine fields. In 2021, we published 181 original research papers (eight related to COVID-19), 17 reviews, five editorials, 13 Letters to the Editor, and the Japanese and European Societies of Neuroradiology Congress Abstracts. We also published an increasing number of manuscripts related to artificial intelligence applications, which requires special guidance. Thus, we were very happy to publish Wagner et al.’s [1] review in September.

I am delighted to thank all authors who deemed *Neuroradiology* to be the preeminent home for their research efforts and articles. I offer heartfelt thanks to our Section Editors and reviewers for identifying the best science out of voluminous submissions. I thank the Editorial Board members for sharing their ideas and supporting a dynamic peer review process and also welcome our new Editorial Board members. Finally, I thank our Managing Editor, Dr. Lisa Babinec, for facilitating the publishing process alongside the production team and Springer Nature for their support and commitment to publication ethics.

In 2022, we aim to stimulate scientific discussions on emerging neuromedical diagnostic and therapeutic concepts by providing two new features with the support of Prof. Mayank Goyal, Calgary: “Controversies” and “Innovations.” Additionally, we continue to welcome reader suggestions regarding urgent questions that should be discussed in our journal.

Prof. Dr. med. Rüdiger von Kummer

Editor-in-Chief
